# Functions of RNF Family in the Tumor Microenvironment and Drugs Prediction in Grade II/III Gliomas

**DOI:** 10.3389/fcell.2021.754873

**Published:** 2022-02-09

**Authors:** Jingwei Zhang, Zeyu Wang, Hao Zhang, Ziyu Dai, Xisong Liang, Shuwang Li, Xun Zhang, Fangkun Liu, Zhixiong Liu, Kui Yang, Quan Cheng

**Affiliations:** ^1^ Department of Neurosurgery, Xiangya Hospital, Central South University, Changsha, China; ^2^ National Clinical Research Center for Geriatric Disorders, Changsha, China; ^3^ Clinical Diagnosis and Therapy Center for Glioma of Xiangya Hospital, Central South University, Changsha, China; ^4^ Department of Clinical Pharmacology, Xiangya Hospital, Central South University, Changsha, China

**Keywords:** lower-grade glioma, tumor microenvironment, immunotherapy, RING finger proteins, Chemotherapy

## Abstract

Increasing evidence has demonstrated that RING finger (RNF) proteins played a vital role in cellular and physiological processes and various diseases. However, the function of RNF proteins in low-grade glioma (LGG) remains unknown. In this study, 138 RNF family members revealed their role in LGG. The TCGA database was used as the training cohort; two CGGA databases and GSE108474 were selected as external validation cohorts. Patients were grouped into cluster 1 and cluster 2, both in the training and validation cohorts, using consensus clustering analysis. The prognosis of patients in cluster 1 is significantly better than that in cluster 2. Meanwhile, biofunction prediction was further introduced to explore the potential mechanisms that led to differences in survival outcomes. Patients in Cluster 2 showed more complicated immunocytes infiltration and highly immunosuppressive features than cluster 1. Enrichment pathways such as negative regulation of mast cell activation, DNA replication, mismatch repair, Th17 cell differentiation, antigen processing and presentation, dendritic cell antigen processing and presentation, dendritic cell differentiation were also enriched in cluster 2 patients. For the last, the main contributors were distinguished by employing a machine learning algorithm. A lot of targeted and small molecule drugs that are sensitive to patients in cluster 2 were predicted. Importantly, we discovered TRIM8, DTX2, and TRAF5 as the most vital contributors from the RNF family, which were related to immune infiltration in LGG tumor immune landscape. In this study, we demonstrated the predicted role of RNF proteins in LGG. In addition, we found out three markers among RNF proteins that are closely related to the immune aspects of LGG, which might serve as novel therapeutic targets for immunotherapy in the future.

## Introduction

Diffuse gliomas, including LGG and glioblastomas (GBM), are the most common malignant tumors among adults in the central nervous system (CNS) (Miller et al., 2019). To data, maximum safe surgical resection, radiotherapy, and chemotherapy remain the mainstay of therapeutic methods for gliomas (Zhang et al., 2019; Funakoshi et al., 2021). However, the prognosis of malignant and invasive gliomas is still far from satisfactory, even with recent improvements in diagnosis and treatment methods (Tan et al., 2020; McKinnon et al., 2021). For example, the median overall survival (OS) of LGG patients is less than 2 years, whereas 5-year survival rate of GBM patients is only about 5% (Alexander and Cloughesy, 2017; An et al., 2020; Zhang et al., 2021a). In addition, several clinicopathological and molecular features determine the outcome of patients with gliomas, such as WHO grade, isocitrate dehydrogenase 1/2 (IDH1/2) mutations, 1p/19q co-deletion, MGMT promoter methylation, subtype (Reifenberger et al., 2017; Molinaro et al., 2019). Nowadays, increasing evidence indicates that tumor immunotherapy focusing on blocking immune checkpoints has achieved remarkable benefits, providing a promising direction for glioma patients (Hodges et al., 2017; Aslan et al., 2020).

The tumor microenvironment (TME) is a highly dynamic and complex ecosystem consisting of tumor cells, stromal cells, extracellular matrix, and various cellular molecules (Giraldo et al., 2019; Baghban et al., 2020). Tumors exhibit immunosuppression and immune evasion through immune checkpoints secreted from stromal cells or tumor cells in the TME, resulting in tumor growth and metastasis. Inhibitors and vaccines targeting classical immune checkpoint molecules in the TME, such as programmed cell death-1 (PD-1) and cytotoxic T-lymphocyte-associated antigen 4 (CTLA-4), have achieved remarkable progress in several types of cancers (Wang et al., 2019; Andrews et al., 2021). Our previous study showed that upregulated CTLA-4 expression was associated with a worse prognosis in glioma (Liu et al., 2020; Zhang et al., 2021b). Meanwhile, the infiltrated immune cells such as tumor-associated macrophages (TAMs), dendritic cells (DCS), natural killer cells (NK), and regulatory T cells (Tregs) in the TME also participate in every step of tumor immune progression (Galli et al., 2020; Li et al., 2021). The glioma tumor microenvironment has proved to play a significant role in promoting angiogenesis, immunosuppression, migration, tumor metastasis, and drug resistance (De Vleeschouwer et al., 2017; Simon et al., 2020; Liu et al., 2021).

The RNF proteins are a group of transmembrane proteins containing a unique three-dimensional domain which is consists of C3HC4 amino acid residues with eight conserved cysteine and histidine residues that combine two zinc cations (Cham et al., 2017; Cham et al., 2018; Kuhns et al., 2020). Most RNF proteins act as E3 ubiquitin ligases and regulate the ubiquitination of membrane proteins under physiological conditions (Campbell et al., 2012). In addition, studies revealed that transmembrane RNF proteins play an essential role in many organelles and cellular progress, including protein transportation, cell proliferation, differentiation, apoptosis, immunomodulatory and mitochondrial dynamics (Amal et al., 2019; Wei et al., 2019). However, in recent years, more and more studies have started to explore the function of RNF proteins in oncogenesis and tumor metastasis (Wang et al., 2016; Liu et al., 2018). For example, Rong Geng et al. (Geng et al., 2017) found that the elevated RNF183 protein in tumor samples promotes the migration and metastasis of colorectal cancer cells through activating the NF-κB-IL-8 axis. Moreover, the overexpressed RNF38 was found to inhibit the expression of neuroblast differentiation-associated protein (AHNAK) and activate the transforming growth factor-β (TGF-β) signaling pathway through ubiquitinating, which is associated with the poor outcome and high recurrence rate of hepatocellular carcinoma patients (Peng et al., 2019). However, the role of RNF proteins in LGG remains largely unclear.

Therefore, we analyzed the clinical and RNA-sequencing data of LGG patients from three different datasets to clarify the whole aspects of RNF proteins in the LGG tumor microenvironment.

## Materials and Methods

### Data Collection

We collected the clinical and transcriptomic data of LGGs from the TCGA (http://cancergenome.nih.gov/), the CGGA-array (Fang et al., 2017) (mRNA microarray database), the CGGA-sequence (Zhao et al., 2017) (mRNA sequencing database) (http://www.cgga.org.cn) and Rembrandt datasets (also known as GSE108474) (Gusev et al., 2018). The Gliovis data portal predicted the (Bowman et al., 2017) subtype of LGG. RNF family members are downloaded from the HGNC database (https://www.genenames.org/).

### Consensus Clustering Analysis

The intersection between RNF family members and gene lists from the TCGA and CGGA databases is performed. Then consensus cluster analysis is introduced with the R package “ConsensusClusterPlus” (Wilkerson and Hayes, 2010) based on data from the TCGA dataset. Parameters of cluster model are set as, distance = “Pearson,” maxK = 10, reps = 1000, pItem = 0.8, pFeature = 1, clusterAlg = “kmdist,” corUse = “complete.obs.” PCA diagram shows the classification of the cluster model.

### Immunogenicity Evaluation

ESTIMATE (Yoshihara et al., 2013) algorithm is applied to calculate the immune score, stromal score, and tumor purity. CIBERSORT (Newman et al., 2019) and xCell (Aran et al., 2017) algorithms are used to show the infiltration ratio of immunocytes. The expression profile of immune escape-associated genes from previous work is mapped with a boxplot. Two types of immunogram (Karasaki et al., 2017; Kobayashi et al., 2020) from previous works are reconstructed based on the “ssgsea” algorithm and shown with radar diagram, boxplot, and heatmap.

### Bio Function Analysis

The Gene Ontology (GO) and Kyoto Encyclopedia of Genes and Genomes (KEGG) based on Gene Set Enrichment Analysis (GSEA) and Gene Set Variation Analysis (GSVA) were used to exploring enrichment signaling pathways between clusters or groups.

### Main Contributor Identification

Main contributors of the cluster model from RNF family members are identified by employing multiple machine learning methods. First, univariate Cox regression analysis and LASSO regression analysis, as previously described, are performed to determine LGG prognosis-associated markers. Then, the xgboost algorithm is performed with the R package “xgboost.” For the last, the Boruta algorithm is introduced to label family members with “Confirmed” or “Rejected” based on the cluster model, and “Confirmed” markers are filtered out. Finally, the Venn diagram shows the intersection of results, which are also selected as leading contributors, from those three methods.

### Potential Compounds Prediction

Drug sensitivity from PRISM and CTRP database and cell line expression profile from CCLE database is integrated to predict potential sensitivity compounds based on the cluster model. Preparation of drug sensitivity matrix and cell line expression matrix is performed as previous work stated. R package “pRRophetic” is used for potential compounds prediction. Similar strategies are also applied to identify compounds from the CellMiner database.

### Statistical Analysis

The Wilcox test was used to compare two groups. In addition, the Kaplan-Meier analysis was applied to analyze the survival prognosis between two groups, and log-rank was used for examination. The ROC curve and corresponding AUC were generated by using the R package “timeROC.” * *p*-value < 0.05, ** *p*-value < 0.01, *** *p*-value < 0.001, and *p*-value < 0.05 is significantly statistical. All analyses were performed with R (version 3.6.1).

## Results

### Clustering Model Based on RNF Genes Identified Two Clusters with Distinct Outcomes and Clinicopathological Features

**FIGURE 1:**
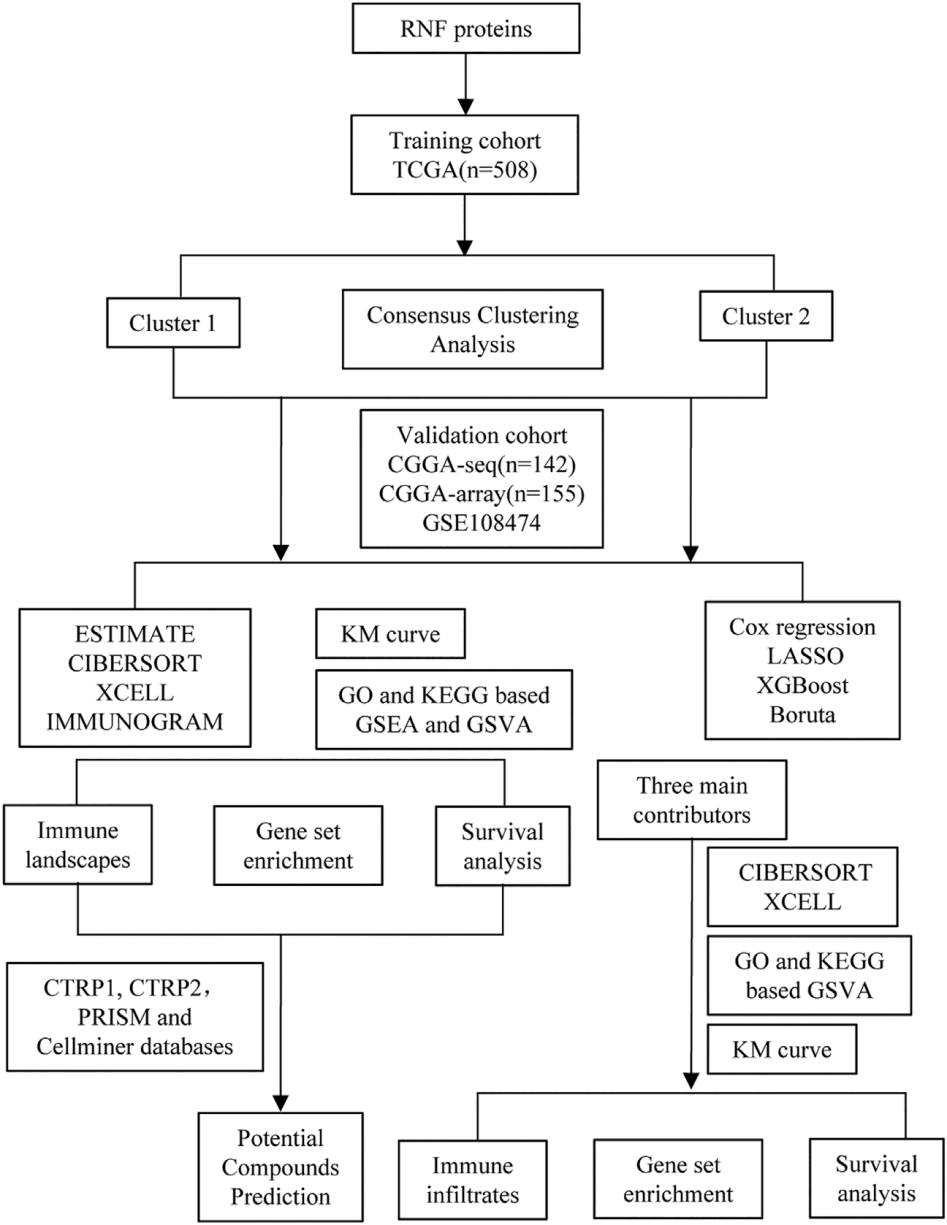
The flow chart of the entire study.

**FIGURE 2:**
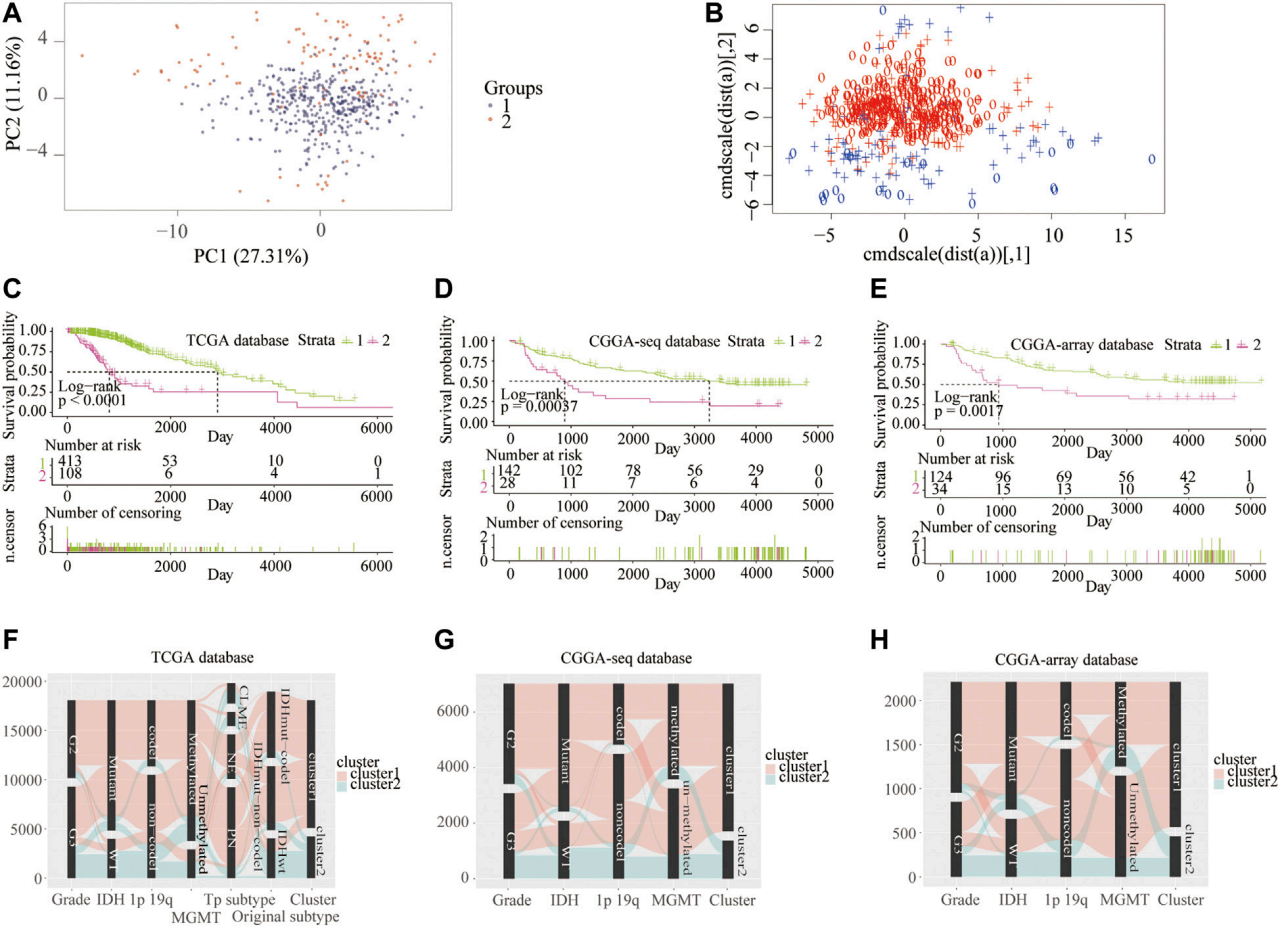
Consensus clustering of samples into cluster 1 and cluster 2 from the training and validation cohorts. Consensus clustering patients into two clusters using principal component **(A)** and support vector machine analysis **(B)** from the TCGA database. Kaplan–Meier overall survival curve between two clusters from the TCGA **(C)**, CGGA-sequence **(D)**, and CGGA-array **(E)** databases. Sankey diagram shows LGG clinicopathologic features between two clusters from the TCGA **(F)**, CGGA-sequence **(G)**, and CGGA-array **(H)** databases.

First, to clarify the prognostic role of RNF proteins in LGG, a total of 138 RNF family members were selected from the public database-GENECARDS (Supplementary Table S1). The flow chart of the entire study is shown in Figure 1, we used the TCGA as the training set, and two CGGA and the Rembrandt (GSE108474) datasets were treated as validation sets. The clinicopathological characteristics of four public databases are shown in Supplementary Table S2. Based on the clustering analysis, the optimal number of clusters was 2 (Supplementary Figure S1A). Then, patients in the TCGA database were divided into two subgroups through the consensus clustering method (Figures 2A,B). Kaplan-Meier analysis in the TCGA database between two clusters showed that patients in cluster 1 had a better prognosis than cluster 2 during the overall survival time (*p* < 0.0001; Figure 2C). Meanwhile, in the CGGA sequence (*p* < 0.001; Figure 2D) and CGGA array (*p* < 0.01; Figure 2E) database, the patients in cluster 1 also had better results than cluster 2 during the overall survival time. Moreover, the patients in cluster 1 also had a better outcome than cluster 2 during the overall survival time in the GSE108474 dataset (Supplementary Figure S1B). We then calculated the AUC of the cluster model in the TCGA dataset (Supplementary Figure S1C), which is 0.74. However, the AUC in three validation sets is 0.61 (CGGAseq; Supplementary Figure S1D), 0.65 (CGGAarray; Supplementary Figure S1E), 0.64 (GSE108474; Supplementary Figure S1F). We thought that the AUC in these validation datasets was lower than 0.7 resulting from the number of samples in validation sets being less than that in the training set. In addition, the Sankey diagram in the TCGA database showed that patients in cluster 1 tend to exhibit favorable clinicopathologic features: IDH mutation and IDH mut-codel subtype (Figure 2F), which were also verified in CCGA-sequence (Figure 2G) and CCGA-array (Figure 2H) databases. These results indicated that the expression of RNF genes is associated with patient’s prognosis in low-grade gliomas and might have a close relationship with the IDH status.

### Functional Enrichment Analysis Between Two Clusters

**FIGURE 3:**
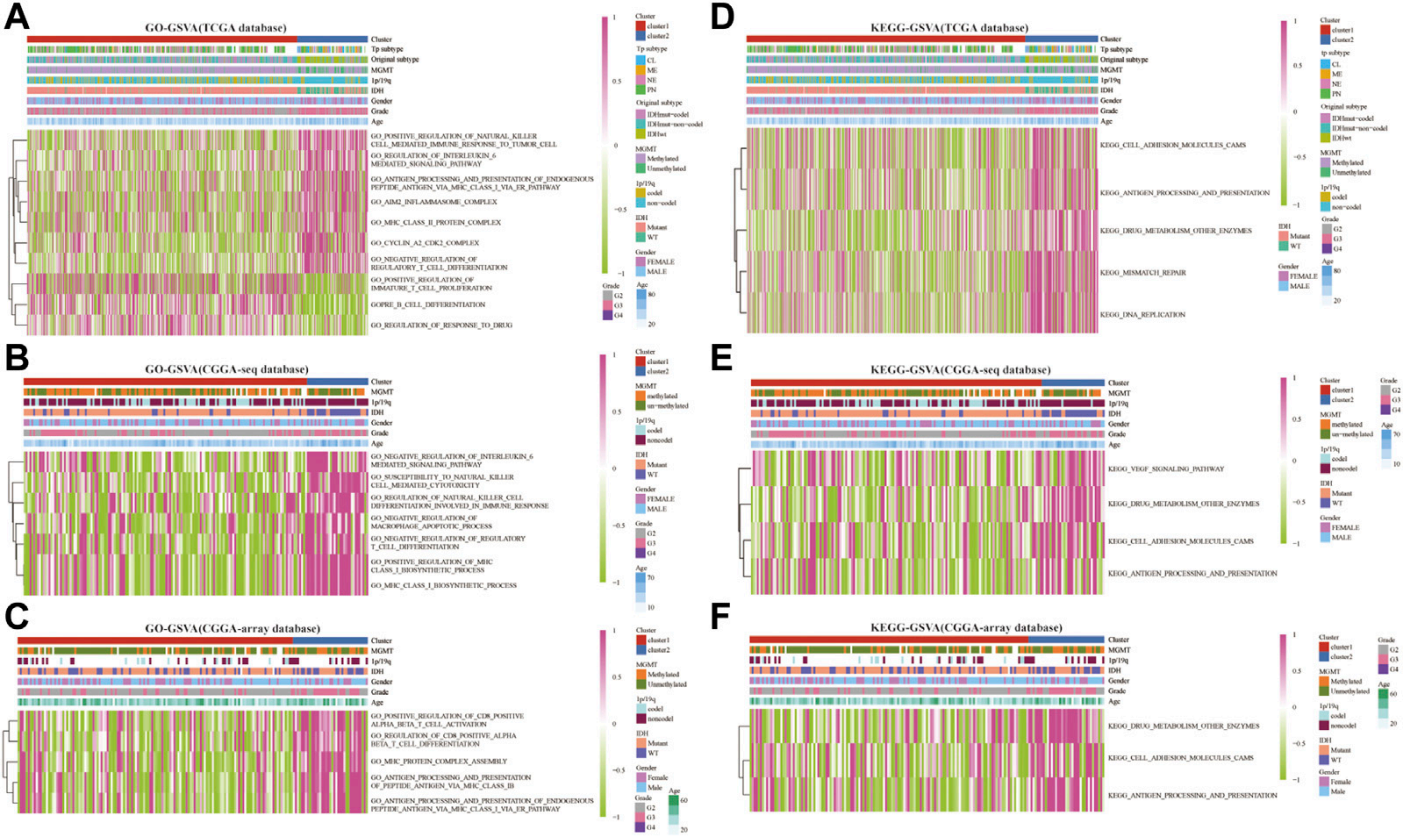
RNF proteins-related biological functions in cluster 1 and cluster 2 from the training and validation cohorts. Gene set variation analysis in cluster 1 and cluster 2 based on GO database from the TCGA **(A)**, CGGA-sequence **(B)**, and CGGA-array **(C)** databases. Gene set variation analysis in cluster 1 and cluster 2 based on the KEGG database from the TCGA **(D)**, CGGA-sequence **(E)**, and CGGA-array **(F)** databases.

Next, we analyzed the related enrichment signaling pathways between clusters 1 and 2 using GO and KEGG-based GSEA and GSVA. GO analysis in the TCGA database showed that several signaling pathways related to immune response were enriched in cluster 2, including positive regulation of natural killer cell-mediated immune response to the tumor cell, negative regulation of IL-6 and mast cell activation, regulation of antigen processing, and presentation, inflammasome and MHC class Ⅱ protein complex, cyclin A2/CDK2 complex, negative regulation of regulatory T cell differentiation (Figure 3A and Supplementary Figure S2A). Meanwhile, the pathways of positive regulation of immature T cell proliferation, B cell differentiation, and regulation of response to the drug were enriched in cluster 1. Furthermore, GO analysis in the CGGA-sequence database showed that the pathways of susceptibility to natural killer cell-mediated cytotoxicity, negative regulation of IL-6, negative regulation of T cell differentiation and macrophage apoptotic process, positive regulation of MHC class Ⅰ biosynthetic process were enriched in cluster 2 (Figure 3B and Supplementary Figure S2B). In addition, GO analysis in the CGGA-array showed that several immune-related pathways, such as positive regulation of CD8^+^αβT cell activation and differentiation, antigen processing, and presentation of peptide antigen *via* MHC classⅠbiosynthetic process (Figure 3C and Supplementary Figure S2C). Furthermore, KEGG analysis indicated that the pathways such as antigen processing and presentation, DNA replication, drug metabolism, other enzymes, cell adhesion molecules CAMs were enriched in cluster 2 in the training and validation databases (Figures 3D–F and Supplementary Figures S2D–F).

### Immune Infiltration Analysis Between Two Clusters

**FIGURE 4:**
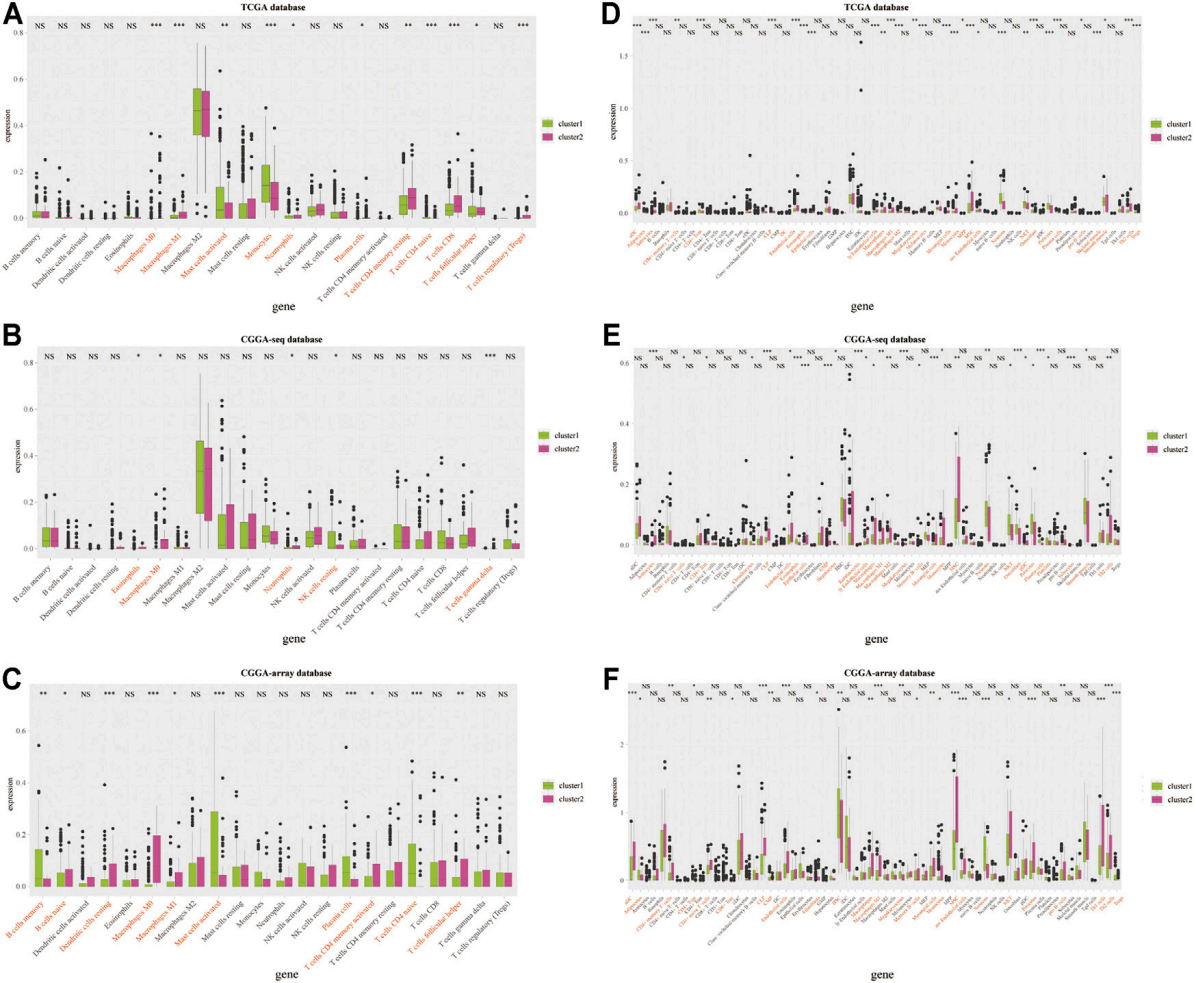
Infiltrated immune cells in cluster 1 and cluster 2 from the training and validation cohorts. Immune infiltrates in two clusters based on the CIBERSORT algorithm from the TCGA **(A)**, CGGA-sequence **(B)**, and CGGA-array **(C)** databases. Immune infiltrates in two clusters based on the xCELL algorithm from the TCGA **(D)**, CGGA-sequence **(E)**, and CGGA-array **(F)** databases. *p < 0.05, **p < 0.01, ***p < 0.001, NS, no significant differences.

Therefore, we examined the immune aspects in the LGG TME between cluster 1 and cluster 2. The results from the TCGA database demonstrated that the expression of TME immune cells in cluster 1 was significantly different from that in cluster 2, including M1 macrophages, monocytes, plasma cells, CD4 memory T cells, and Tregs (*p* < 0.05; Figures 4A,D and Supplementary Figures S3A,D). The immune cell types infiltrated in cluster 1 and cluster 2 from the CGGA sequence database were also significantly different, such as eosinophils, macrophages, and NK cells (*p* < 0.05; Figures 4B,E and Supplementary Figures S3B,E). Results from the CGGA-array database showed that large amounts of immune cells in the TME were different from cluster 1 and cluster 2, including memory B cells, dendritic cells, M1 macrophages, activated CD4 memory T cells, and follicular helper T cells (*p* < 0.05; Figures 4C,F and Supplementary Figures S3C,F). Moreover, the tumor purify was higher in cluster 1, whereas the estimated score, stromal score, and immune score were lower in cluster 1, both in the TCGA (*p* < 0.001; Supplementary Figure S4A), CGGA-sequence (*p* < 0.05; Supplementary Figure S4B) and CGGA-array (*p* < 0.05; Supplementary Figure S4C) databases.

### Immunosuppressive Aspects Analysis Between Two Clusters

**FIGURE 5:**
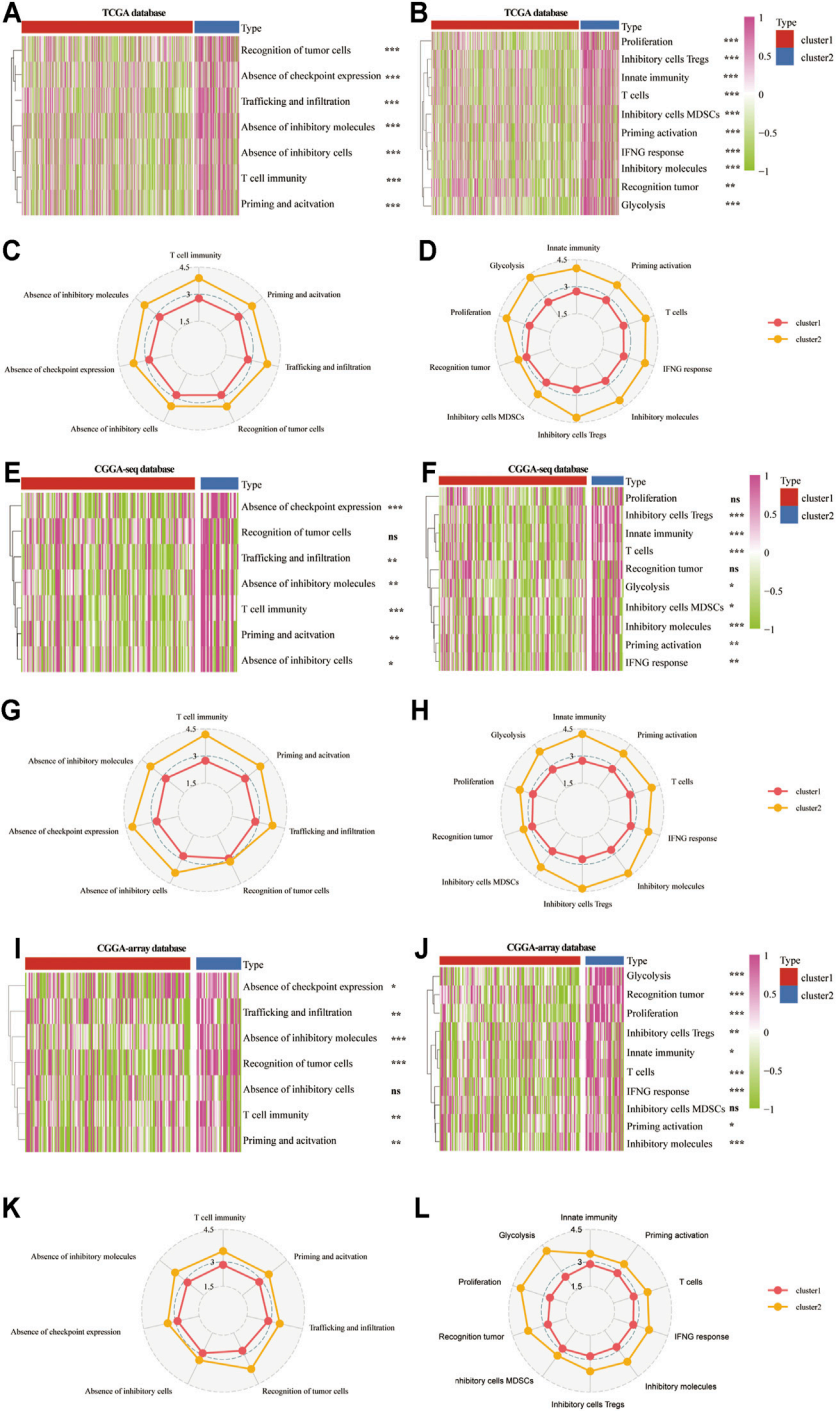
The patient-specific landscape of the LGG tumor microenvironment in cluster 1 and cluster 2 from the training and validation cohorts. Heatmap shows the expression of immune landscapes in two clusters based on 2017 (left) and 2020 (right) immunogram algorithm from the TCGA **(A**,**B)**, CGGA-sequence **(E**,**F)**, and CGGA-array **(I**,**J)** databases. The radar chart shows the expression of the immune landscape in two clusters based on the 2017 (left) and 2020 (right) immunogram algorithm from the TCGA **(C**,**D)**, CGGA-sequence **(G**,**H)**, and CGGA-array **(K**,**L)** databases. *p < 0.05, **p < 0.01, ***p < 0.001, NS, no significant differences.

Then, we analyzed the patient-specific landscapes of the tumor microenvironment in the LGG using two types of immunogram (2017, 2010). Results showed that several immunosuppressive progresses, including the absence of checkpoint expression, trafficking, and infiltration, absence of inhibitory molecules, T cell immunity, priming, and activation, were significantly enriched in cluster 2 from the TCGA (*p* < 0.001; Figures 5A,C), CGGA-sequence (*p* < 0.01; Figures 5E,G) and CGGA-array (*p* < 0.05; Figures 5I,K) databases. Meanwhile, other immunological progress, including inhibitory cells Tregs, innate immunity, T cells, glycolysis, inhibitory molecules, priming activation, and IFNG response was also significantly enriched in cluster 2 from the TCGA (*p* < 0.01; Figures 5B,D), CGGA-sequence (*p* < 0.05; Figures 5F,H) and CGGA-array (*p* < 0.05; Figures 5J,L) databases.

**FIGURE 6:**
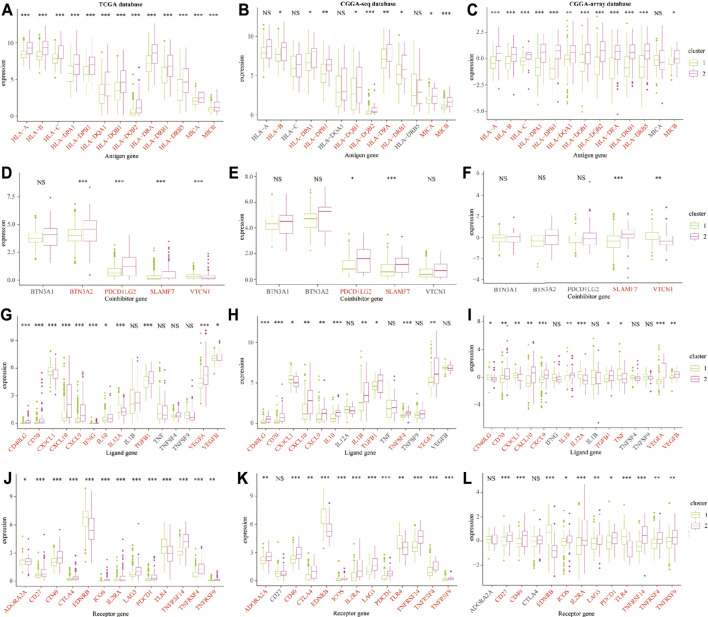
The immune escape-associated gene expression in cluster 1 and cluster 2 from the training and validation cohorts. The expression of antigen genes in cluster 1 and cluster 2 from the TCGA **(A)**, CGGA-sequence **(B)**, and CGGA-array **(C)** databases. The expression of co-inhibitory genes in cluster 1 and cluster 2 from the TCGA **(D)**, CGGA-sequence **(E)**, and CGGA-array **(F)** databases. The expression of ligand genes in cluster 1 and cluster 2 from the TCGA **(G)**, CGGA-sequence **(H)**, and CGGA-array **(I)** databases. The expression of receptor genes in cluster 1 and cluster 2 from the TCGA **(J)**, CGGA-sequence **(K)**, and CGGA-array **(L)** databases. *p < 0.05, **p < 0.01, ***p < 0.001, NS, no significant differences.

Furthermore, we explored the expression profiles of immune escape-associated genes between cluster 1 and cluster 2 from the training and validation databases. Results indicated that antigen genes such as HLA-B, HLA-DPA1, HLA-DPB1, HLA-DQB2, HLA-DRA, HLA-DRB1, and MICB were upregulated in cluster 2 compared to cluster 1 from the three public databases (*p* < 0.05; Figures 6A–C). The expression of the co-inhibitory gene-SLAMF7 was higher in cluster 2 (*p* < 0.05; Figures 6D–F). The levels of ligand genes, including CD40LG, CD70, CXCL10, CXCL9, IL-10, TGFB1, and CEGFA, were higher in cluster 2, whereas the expression of CX3CL1 was lower in cluster 2 (*p* < 0.05; Figures 6G–I). The levels of receptor genes, including CD40, ICOS, IL2RA, LAG3, PDCD1, TNFRSF14, TNFRSF4, and TNFRSF9, were increased in cluster 2, whereas the expression of EDNRB and TLR-4 was lower in cluster 2 (*p* < 0.05; Figures 6J–L). In addition, the levels of cell adhesion genes, including ICAM-1 and ITGB2, were elevated in cluster 2 (*p* < 0.05; Supplementary Figures S5A–C). The expression of costimulatory genes, including CD28 and CD80, were higher in cluster 2 (*p* < 0.05; Supplementary Figures S5D–F). The expression of other genes, such as GZMA and PRF1, were overexpressed in cluster 2 (*p* < 0.05; Supplementary Figures S5G–I). These results demonstrated that the TNF genes played an essential role in the immune infiltration and were related to the immunosuppressive progress in the LGG TME.

### Prediction of Sensitive Drugs

**FIGURE 7:**
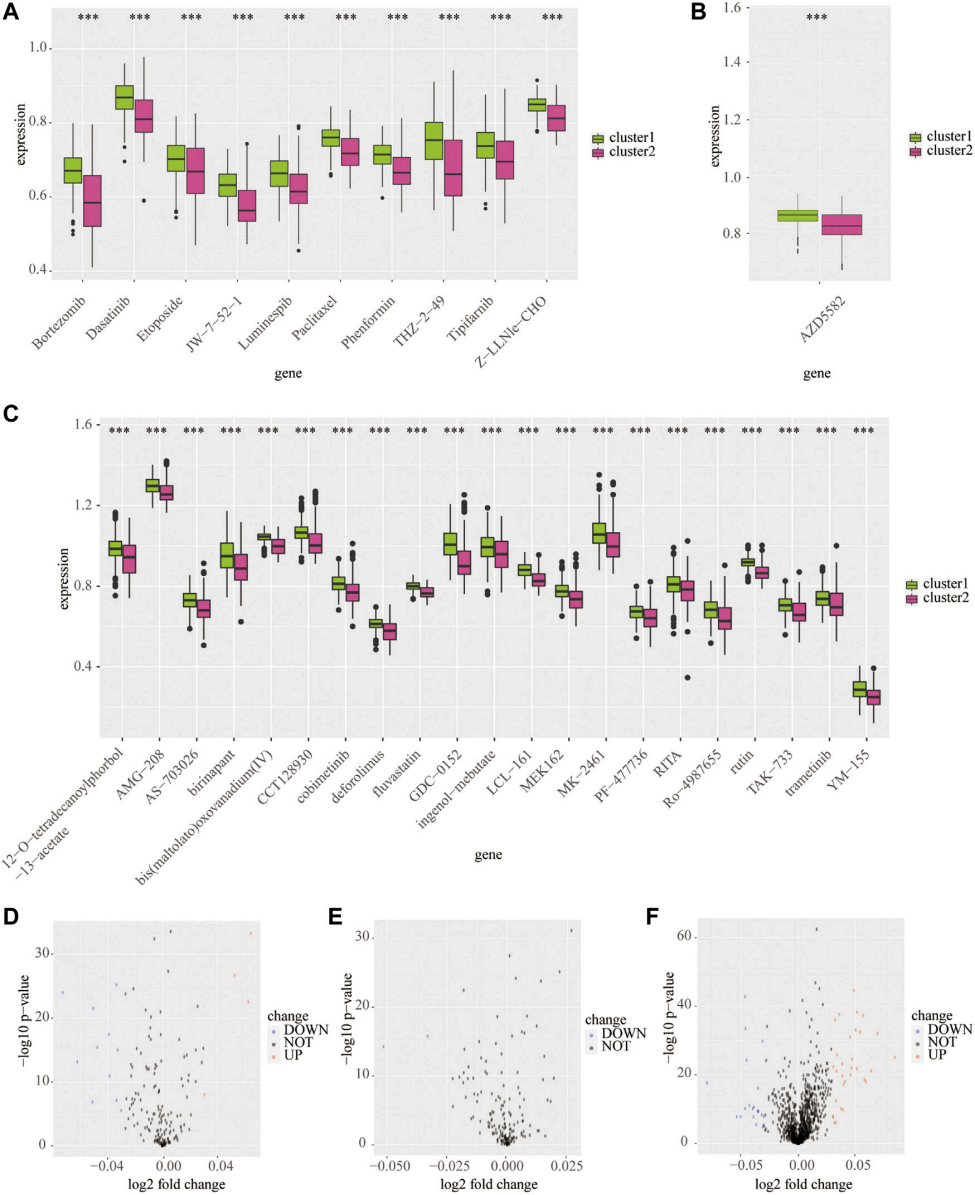
Predicted potential sensitivity compounds based on the cluster model using public databases. The box plot shows sensitivity compounds between cluster 1 and cluster 2 based on the CTRP1 **(A)**, CTRP2 **(B)**, and PRISM **(C)** databases. The volcano plots shows sensitivity compounds between cluster 1 and cluster 2 based on the CTRP1 **(D)**, CTRP2 **(E)**, and PRISM **(F)** databases. ***p < 0.001.

Next, we predicted the sensitive drugs between cluster 1 and cluster 2 from the public databases. The top 50 sensitive drugs between clusters 1 and 2 were exported from the CELLMINIER database (Supplementary Table S3). Data from the CTRP1 database showed that cluster 2 exhibited significantly more sensitivity to bortezomib, dasatinib, JW-7-52-1, phenformin, and THZ-2-49 than cluster 1 (*p* < 0.001; Figures 7A,D). In addition, the CTRP2 database showed that cluster 2 exhibited significant sensitivity to AZD5582 comparing with cluster 1 (*p* < 0.001; Figures 7B,E). In addition, the PRISM database showed that cluster 2 exhibited significant sensitivity to many drugs compared to cluster 1 (*p* < 0.001; Figures 7C,F).

### Main Contributor Identification, Immune Infiltration, and Enrichment Pathway Analysis

**FIGURE 8:**
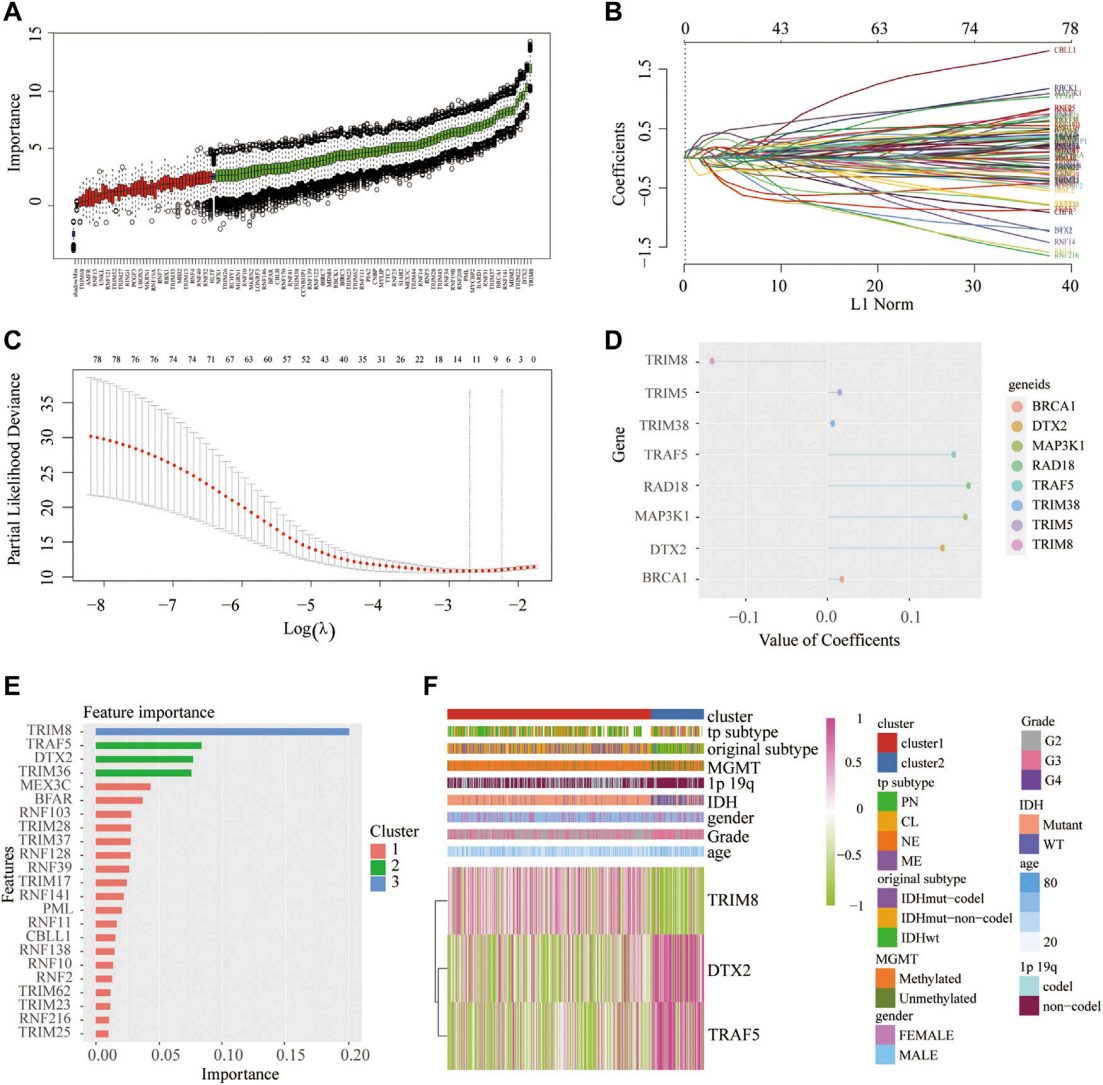
Identification of main contributors of the cluster model from RNF family members. Identify main contributors of the cluster model from RNF family members by Boruta algorithm **(A)**, LASSO regression analysis **(B**–**D)**, and Xgboost algorithm **(E)**. Heatmap shows the expression of three main contributors in two clusters **(F)**.

Finally, we used three machine-learning methods-LASSO, XGBOOST, and BORUTA algorithms to identify the main contributors of the cluster model from RNF family members (Figures 8A–E). The result showed that TRIM8, TRAF5, and DTX2 are the top three contributors. Then, the association of those genes with clinical features was also mapped with heatmap (Figure 8F). The heatmap showed that cluster 2 has higher DTX2 and TRAF5 and a lower expression of TRIM8. Meanwhile, the results showed that the overexpressed DTX2 and TRAF5 were associated with IDH WT status, whereas the overexpressed TRIM8 were associated with IDH mutant status.

**FIGURE 9:**
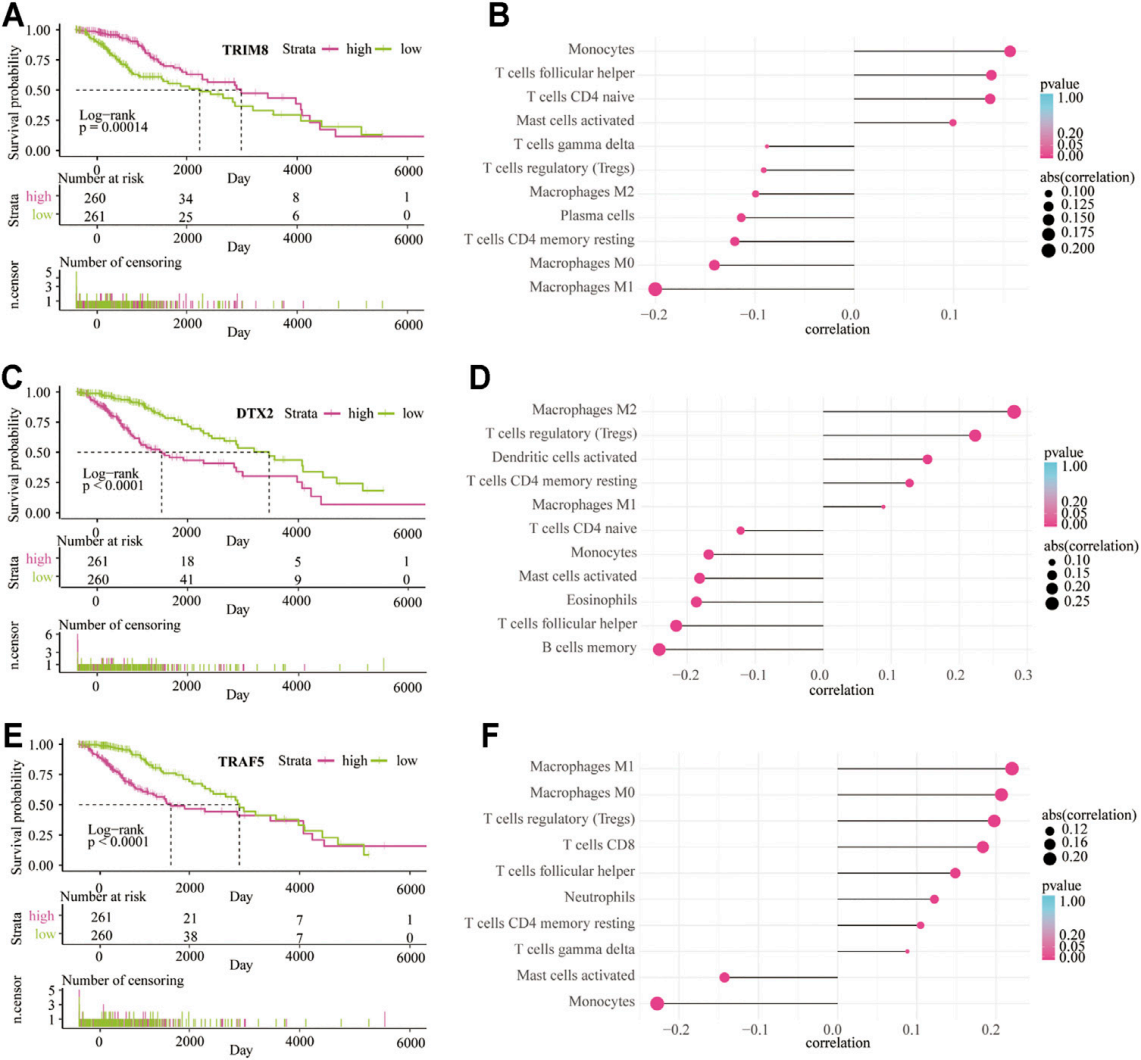
The survival analysis and immune infiltrate are based on the expression of three main contributors. Kaplan–Meier overall survival curve between low-risk and high-risk groups based on the expression of TRIM8 **(A)**, DTX2 **(C)**, and TRAF5 **(E)**. CIBERSORT algorithm shows the relationship between the immune infiltrates and the expression of TRIM8 **(B)**, DTX2 **(D)**, and TRAF5 **(F)**.

Patients in the high expression group of TRIM8 have a better outcome than the low expression group (*p* < 0.001; Figure 9A). On the contrary, patients in the high expression group of DTX2 (*p* < 0.001; Figure 9C) and TRAF5 (*p* < 0.001; Figure 9E) have a worse outcome than the low expression group. These data indicated that the high expression of TRIM8 in the LGGs TME might play a protective role, while the increased expression of DTX2 and TRAF5 in the LGG TME may act as a detrimental role. In addition, the CIBERSORT analysis showed that the most positively correlated cell with TRIM8 is monocyte, and the most negatively correlated cell is M1 macrophage (Figure 9B). The most positively correlated cell with DTX2 is the M2 macrophage, and the most negatively correlated cell is the memory B cell (Figure 9D). The most positively correlated cell with TRAF5 is the M1 macrophage, and the most negatively correlated cell is the monocyte (Figure 9F). These results indicated that the monocyte and memory B cells in the TME might inhibit the LGG progress and contribute to a good outcome. At the same time, the macrophages in the TME may promote the LGG progress and lead to a worse outcome. In addition, we also used xCELL analysis to show the infiltrated immune cells related to the three genes in LGG TME (Supplementary Figure S6). Moreover, the enriched signaling pathways related to the three genes were also displayed by GO-based GSVA analysis (Supplementary Figure S7).

## Discussion

Low-grade glioma is a group of heterogeneous neoplasms originating from the glial cells nearby neurons and accounts for more than 6% of all primary central nervous system (CNS) tumors in adults (Ostrom et al., 2019). With the rapid development of high-throughput sequencing technology, more and more novel biomarkers related to the prognosis of LGG have been discovered in recent years (Aquilanti et al., 2018; Hsu et al., 2019; Zhang et al., 2020; Zhang et al., 2021c). Recent research focuses on the predictive value, and pathogenic mechanism of RNF proteins have been conducted in several cancer types. For example, the expression level of RNF6 was upregulated in both tumor samples and cell lines of gastric cancer. Furthermore, knockdown of RNF6 significantly increased the cleavage of PARP and promoted cell apoptosis through the SHP-1/STAT3 signaling pathway, which eventually inhibits gastric cancer cell growth (Huang et al., 2018). In another study, RNF121 levels were found decreased in renal cell carcinoma samples than adjacent normal tissues (Zhao et al., 2014). Further research revealed that overexpressed RNF121 inhibited the growth and invasion of human renal cell carcinoma cells by activating NF-κB signaling pathways. However, the relevance between RNF proteins and LGG development remains poorly understood.

In this study, we explore the landscape of RNF proteins in the tumor microenvironment of LGG both from the TCGA and CGGA databases. We established a clustering model based on the expression of RNF proteins and found a significant difference in prognosis between the two clusters. Molecular mutations, especially IDH enzyme mutation status, were associated with the outcome of LGG and GBM. Generally, IDH-mutant gliomas have a longer overall survival time than the IDH wide-type (WT) counterparts (Olar et al., 2015; Youssef and Miller, 2020). In the current study, we found that the status of IDH was different between the two clusters. This result means that RNF proteins might have a close relationship with the IDH mutant status of LGG. These data provide new insight into the mechanisms underlying the RNF proteins upon LGG progression.

Infiltrated immune cells and stromal cells in the TME influenced the tumor’s response to the immune system. For example, upregulated tumor infiltrated CD3^+^ and CD8^+^ were associated with longer survival time with an integrated immunosuppressive system in the tumor microenvironment (Kmiecik et al., 2013). Tumor-associated M1-type macrophages are considered to exbibit pro-inflammatory and anti-tumoral effects, while tumor-associated M2-type macrophages are associated with anti-inflammatory and pro-tumoral functions (Madeddu et al., 2018; Macciò et al., 2020). It is well known that myeloid-derived suppressor cells (MDSCs) can act as the primary mediators of immune responses in many cancers and other pathological progress. Tregs can regulate immune suppression of anti-tumor immune response in the tumor microenvironment (Bronte et al., 2016; Li et al., 2020; Pokhrel et al., 2021). DCS identified and processed tumor-associated antigens in the tumor microenvironment and promoted anti-tumor immunity by modulating other immune cells’ functions (Wculek et al., 2020). In this study, the GO and KEGG analysis indicated that the expression levels of RNF proteins were significantly accompanied by immune cells infiltration and checkpoint expression related signaling pathways in LGG, among which T cell and mast cell activation, DCs antigen processing and differentiation, Th17 cell differentiation, absence of checkpoint expression, inhibitory Tregs and MDSCs were most significant. In addition, the RNF proteins expression was significantly associated with tumor purity, immune score, and stromal score in the LGG TME based on the ESTIMATE algorithm. Various immune cells in the LGG TME were related to the expression of RNF proteins, including macrophages, monocytes, plasma cells, CD4 memory T cells, Tregs, neutrophils, and mast cells. Meanwhile, large amounts of immune checkpoints were found to be different expressed between the two clusters. Taken together, we proposed that RNF proteins may be involved in the regulation of immune response in the LGG TME by recruiting immune cells and regulating the expression of immune checkpoints.

Furthermore, we identified three main contributors among RNF proteins: TRIM8, DTX2, and TRAF5. Results showed that the upregulated expression of DTX2 and TRAF5 were associated with IDH WT status and a poor outcome in LGG. In contrast, the elevated expression of TRIM8 was associated with IDH mutant status and a better prognosis in LGG. Furthermore, CIBERSORT and xCELL algorithms demonstrated that these three genes are essential in recruiting immune cells, such as monocytes, macrophages, T cells follicular helper, CD4 naïve T cells, Tregs, CD8 T cells.

Until right now, effective drugs for IDH WT LGG treatment are still limited. Finally, we discovered various small molecular drugs that exhibited sensitivity to cluster 2, such as bortezomib, dasatinib and phenformin, which have been proved to inhibit the growth of human glioma cells in previous studies (Jantas et al., 2018; Wang et al., 2018; Miklja et al., 2020). At the same time, we also discovered many new drugs sensitive to IDH WT glioma that has not been reported before, including AMG−208, JW−7−52−1, THZ−2−49, AZD5582, and so on. These small molecular drugs might help to improve the treatment effect of IDH WT LGG in the future.

To sum up, we established a clustering model based on the expression of RNF proteins, which can be applied to predict the outcome of LGG patients. In addition, we explored the relationship between the immune aspects in the LGG tumor microenvironment and RNF proteins. Moreover, we found three main contributors among RNF proteins that were closely associated with LGG progress. Importantly, we explored lots of sensitive drugs, which might help to improve the treatment effect of patients with LGG in the future. However, there are some limitations to this study. First of all, only public data was used for analysis in this study, which has not been verified with our data. Meanwhile, the fundamental function of RNF genes in regulating immune cells infiltration and checkpoints expression in the LGG TME was not explored through *in vivo* and *in vitro* studies. Secondly, we identified three key markers, but these three genes’ role in LGG is still far from discovered in this paper. The specific mechanisms of RNF genes involved in LGG immunity need further exploration.

## Data Availability

The original contributions presented in the study are included in the article/Supplementary Material, further inquiries can be directed to the corresponding authors.

## Supplementary Material

Supplementary Figure S1 Clustering analysis and predictive ability of the risk signature in the four datasets. Cluster analysis to obtain the best number of clusters in the TCGA **(A)**. Kaplan—Meier overall survival curve between two clusters from the Rembrandt dataset **(B)**. The AUC of the cluster model in the TCGA **(C)**, CGGA-sequence **(D)**, CGGA-array **(E)**, and Rembrandt **(F)** databases.

Supplementary Figure S2 Enrichment of signaling pathways in cluster 2 from the training and validation cohorts. Gene set enrichment analysis in cluster 2 based on G.O. database from the TCGA **(A)**, CGGA-sequence **(B)**, and CGGA-array **(C)** databases. Gene set enrichment analysis in cluster 2 based on the KEGG database from the TCGA **(D)**, CGGA-sequence **(E)**, and CGGA-array **(F)** databases.

Supplementary Figure S3 Immune infiltrates in cluster 1 and cluster 2 from the training and validation cohorts. Heatmap shows infiltrated immune cells in two clusters based on the CIBERSORT algorithm from the TCGA **(A)**, CGGA-sequence **(B)**, and CGG-aarray **(C)** databases. In addition, the heatmap shows infiltrated immune cells in two clusters based on the xCELL algorithm from the TCGA **(D)**, CGGA-sequence **(E)**, and CGGA-array **(F)** databases.

Supplementary Figure S4 Immune landscapes in cluster 1 and cluster 2 from the training and validation cohorts. The tumor purity, estimate score, immune score, stromal score in cluster 1 and cluster 2 from the TCGA **(A)**, CGGA-sequence **(B)**, and CGGA-array **(C)** databases. **p* < 0.05, ***p* < 0.01, ****p* < 0.001.

Supplementary Figure S5 Levels of immune escape associated genes in cluster 1 and cluster 2 from the training and validation cohorts. The expression of cell adhesion genes in cluster 1 and cluster 2 from the TCGA **(A)**, CGGA-sequence **(B)**, and CGGA-array **(C)** databases. The expression of costimulatory genes in cluster 1 and cluster 2 from the TCGA **(D)**, CGGA-sequence **(E)**, and CGGA-array **(F)** databases. The expression of other genes in cluster 1 and cluster 2 from the TCGA **(G)**, CGGA-sequence **(H)**, and CGGA-array **(I)** databases. **p* < 0.05, ***p* < 0.01, ****p* < 0.001, NS: no significant differences.

Supplementary Figure S6 Infiltrated immune cells are based on the expression of three main contributors. XCELL algorithm shows the relationship between the immune infiltrates and the expression of TRIM8 **(A)**, DTX2 **(B)**, and TRAF5 **(C)**.

Supplementary Figure S7 Enrichment of signaling pathways based on the expression of three main contributors. GO enrichment analysis based on GSVA analysis of TRIM8 **(A)**, DTX2 **(B)**, and TRAF5 **(C)**.

Supplementary Table S1 Total RNF family gene list from HGNC database.

Supplementary Table S2 The clinicopathological characteristics of four public databases.

Supplementary Table S3 Top 50 predicted drugs from the Cellminer database.
