# Supplementary figures and images for: Functions of RNF Family in the Tumor Microenvironment and Drugs Prediction in Grade II/III Gliomas
Source: Front Cell Dev Biol. 2022 Feb 9;9:754873. doi: 10.3389/fcell.2021.754873 (PMC8864229; doi:10.3389/fcell.2021.754873)

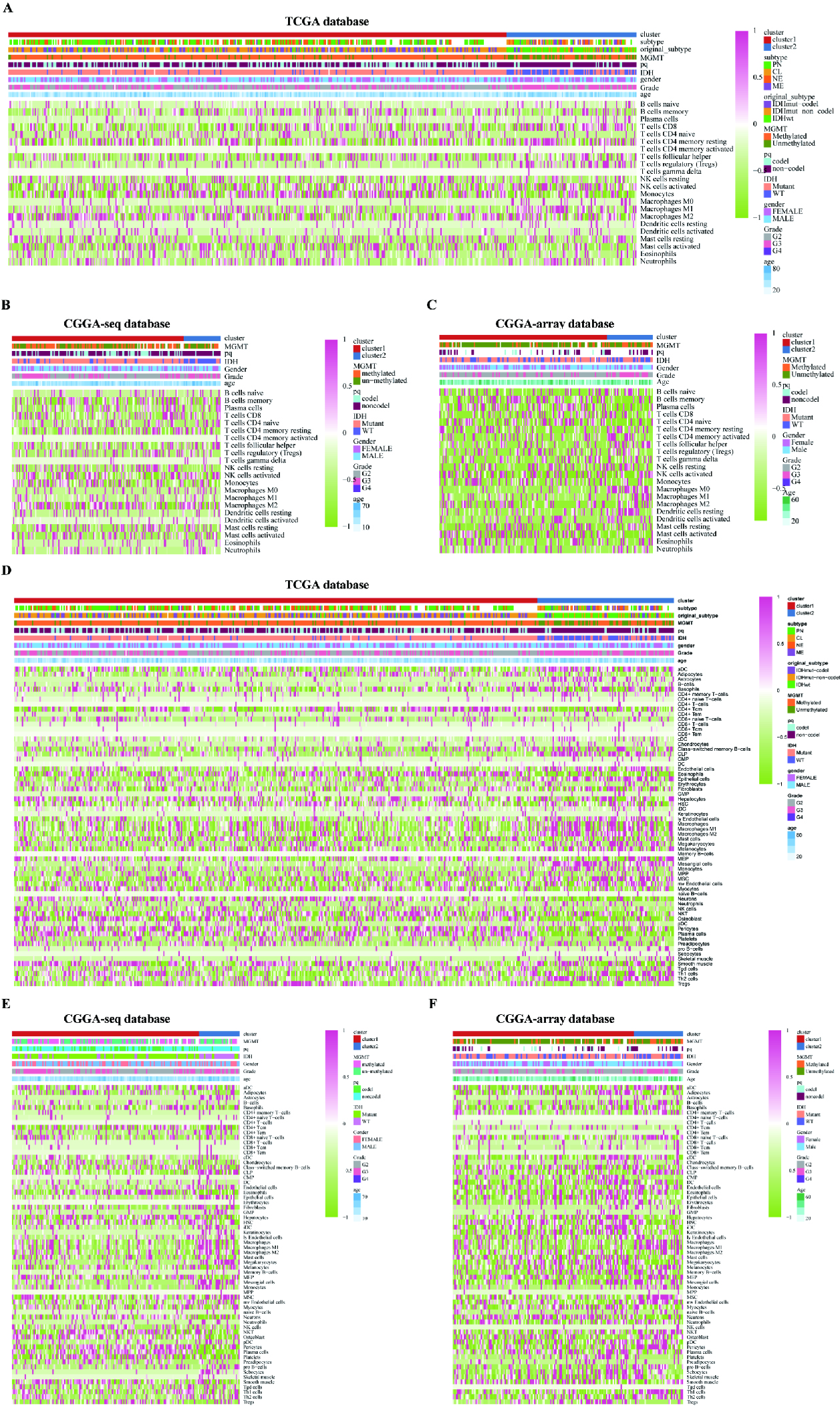

Supplement: Supplementary file 2 [file Image3.JPEG]

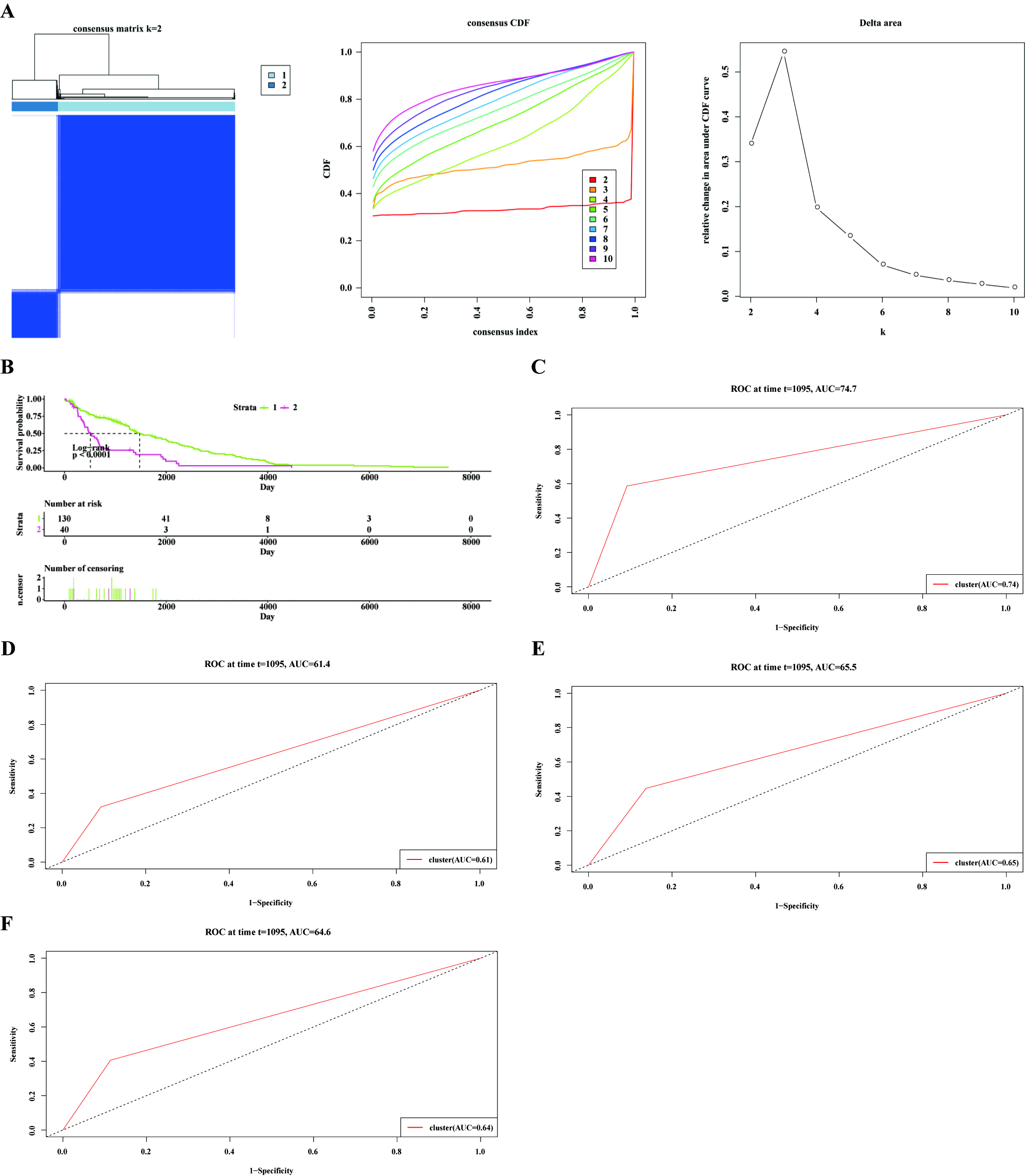

Supplement: Supplementary file 4 [file Image1.JPEG]

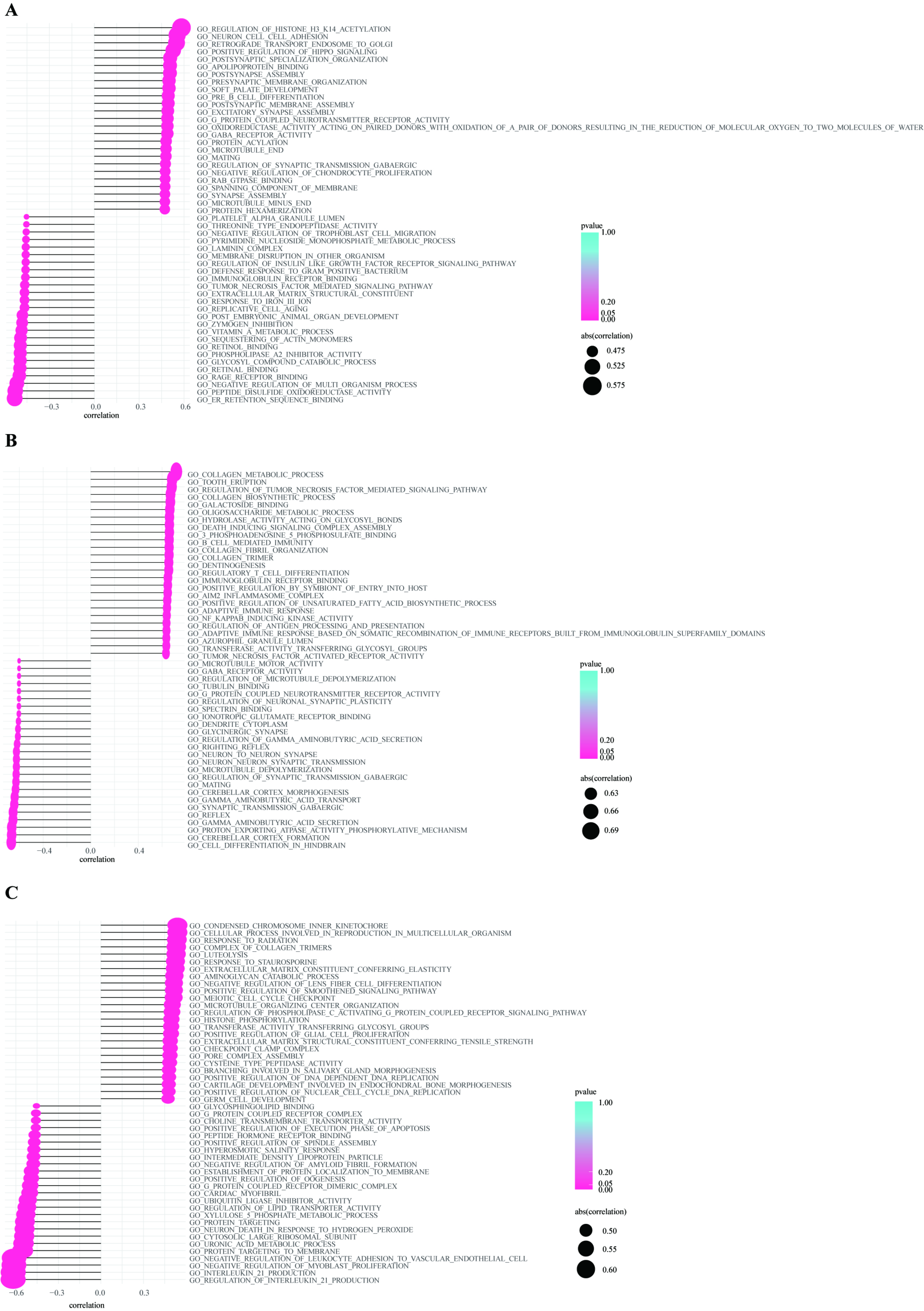

Supplement: Supplementary file 6 [file Image7.JPEG]

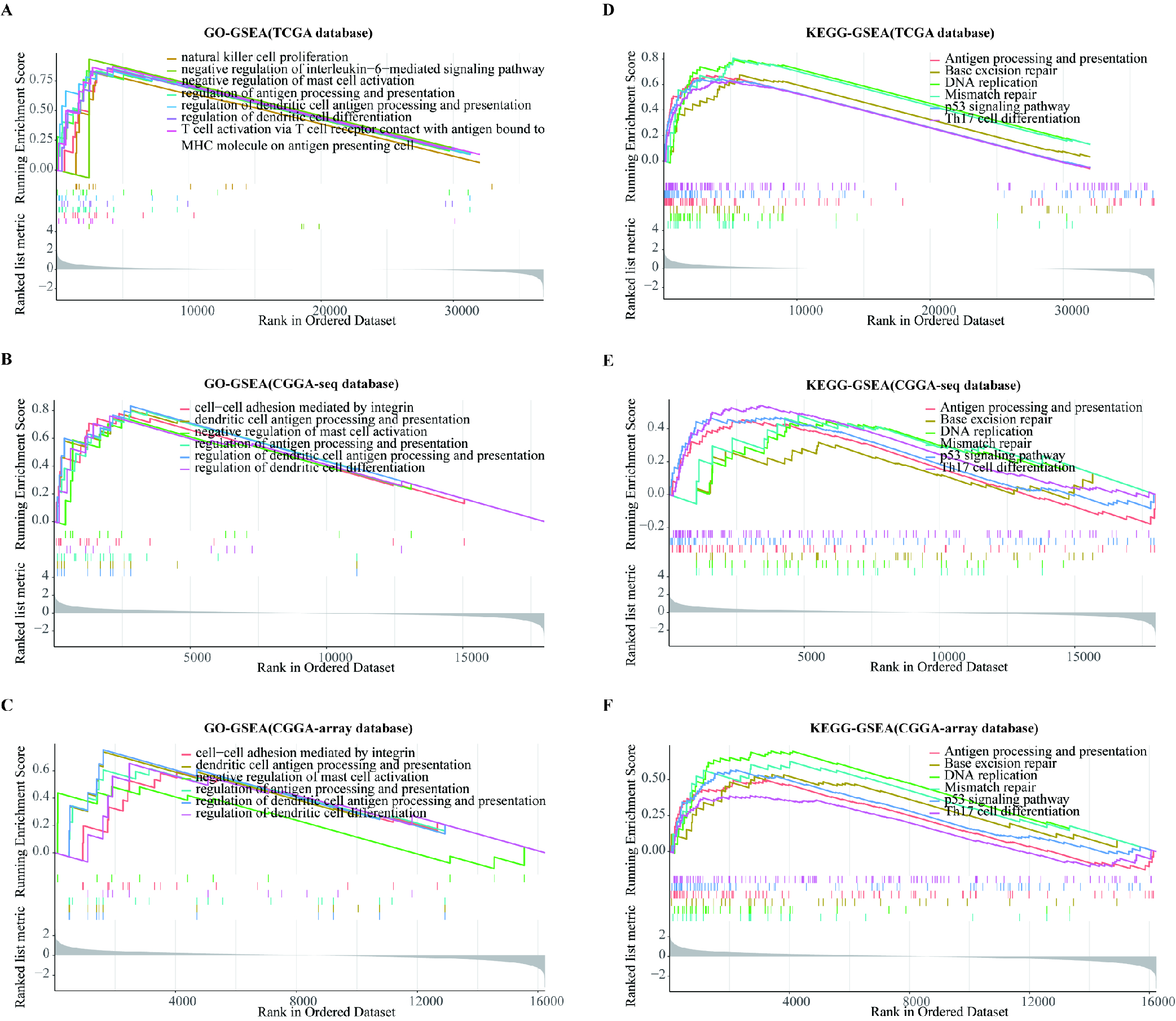

Supplement: Supplementary file 7 [file Image2.JPEG]

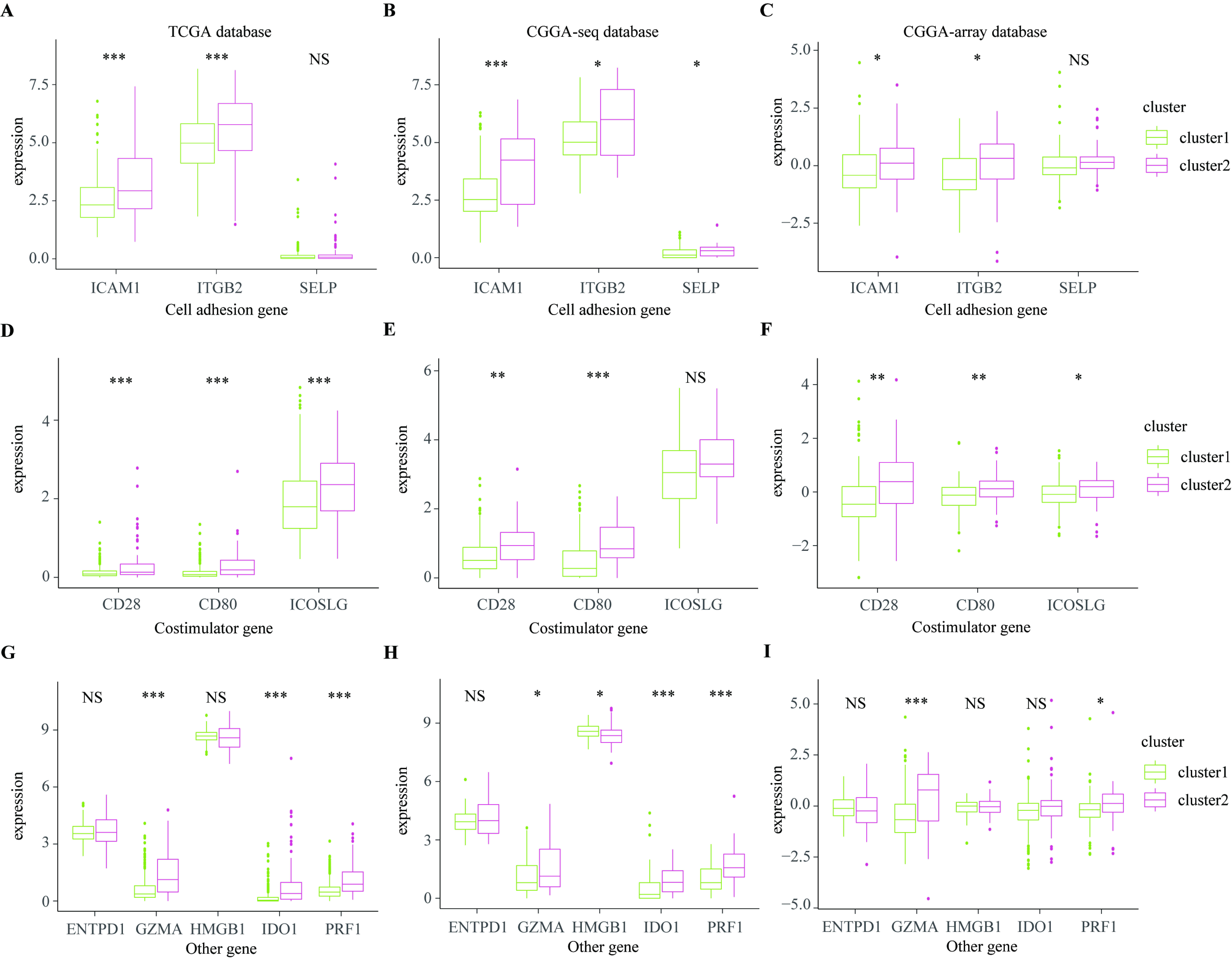

Supplement: Supplementary file 8 [file Image5.JPEG]

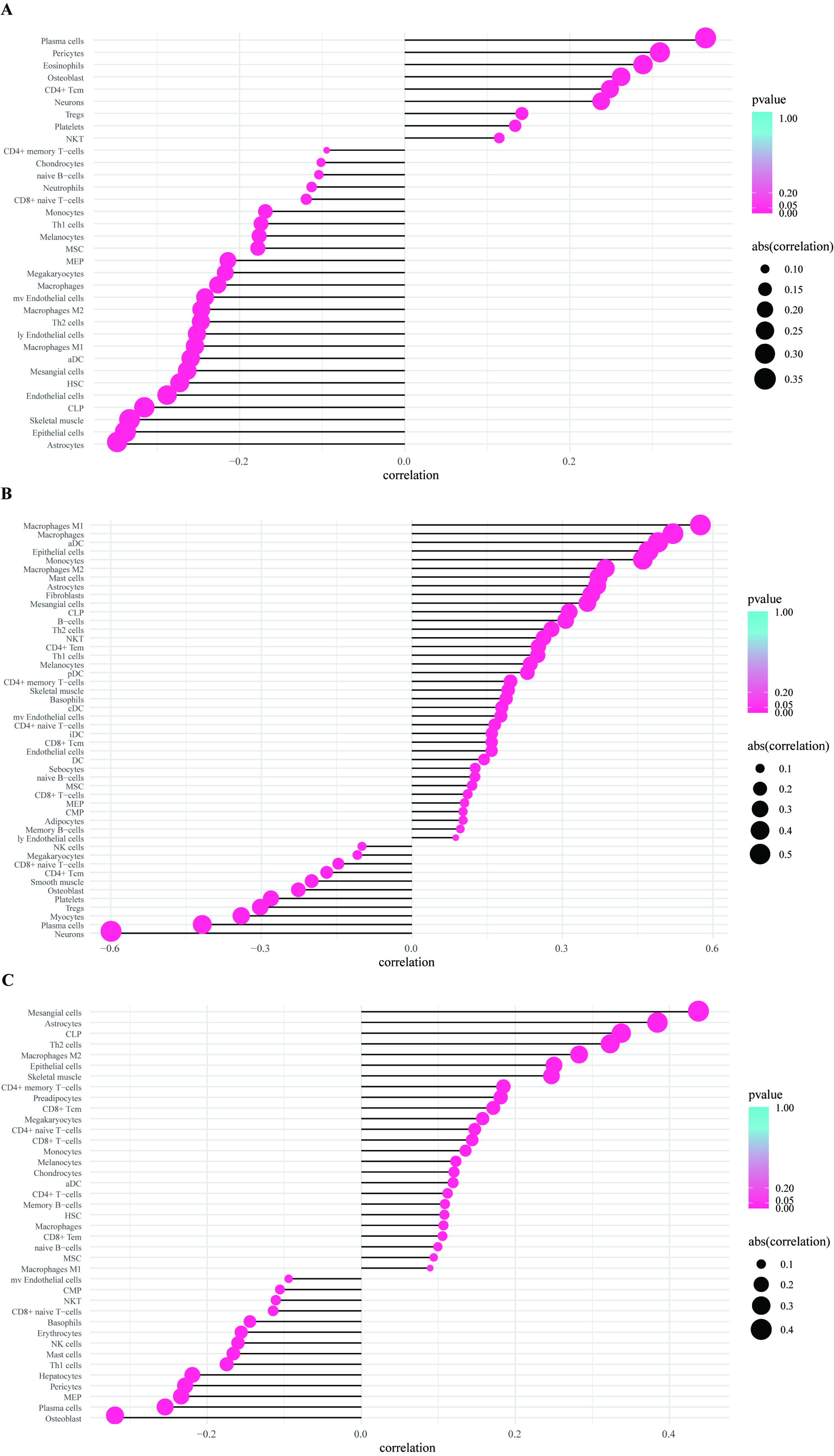

Supplement: Supplementary file 10 [file Image6.JPEG]
